# Prevalence of Onychomycosis in Diabetic Patients: A Case-Control Study Performed at University Hospital Policlinico in Catania

**DOI:** 10.3390/jof8090922

**Published:** 2022-08-30

**Authors:** Laura Trovato, Maddalena Calvo, Rocco De Pasquale, Guido Scalia, Salvatore Oliveri

**Affiliations:** 1 U.O.C. Laboratory Analysis Unit, A.O.U. “Policlinico-San Marco”, 95125 Catania, Italy; 2 Department of Biomedical and Biotechnological Sciences, University of Catania, 95123 Catania, Italy; 3 Unit of Dermatology, Department of General Surgery and Surgical-Medical Specialties, University of Catania, 95124 Catania, Italy

**Keywords:** onychomycosis, diabetes, fungal nail infections, dermatophytes

## Abstract

Diabetes is characterized by an increased rate of serum glucose due to defects in insulin secretion, insulin action or both conditions. Glucose excesses can lead to extended cellular damage, with the consequence of several infectious and non-infectious skin disorders. The aim of the present study was to evaluate the toenail onychomycosis incidence in diabetic patients and healthy ones. The non-interventional, retrospective study was performed at the mycology laboratory of the University hospital “Policlinico-San Marco” in Catania, Italy, for over one year. Nail clippings were collected to perform microscopic and cultural exams, which allowed for the identification of fungal aetiological agents. A total of 715 patients (47 diabetic and 668 non-diabetic patients) were enrolled. In diabetic patients, dermatophytes were the most common cultural isolates (50%), followed by yeasts and moulds in 30.8% and 19.2%, respectively. In non-diabetic patients, the distribution of dermatophytes, yeasts and non-dermatophytic moulds was 67.4%, 5.3% and 27.3%, respectively. According to our results, diabetic patients are more predisposed to nail fungal infection. Our data suggest that dermatological follow-ups should always be performed for diabetic patients. All skin and nail disorders should be carefully monitored to perform a diagnostic confirmation and correct management of diabetic patients.

## 1. Introduction

The diabetic disease is an endocrine disease characterized by an increased rate of serum glucose due to defects in insulin secretion, insulin action or both conditions [1]. All over the world, the number of diabetic patients is dramatically increasing, with a global prevalence nearly doubling since 1980, rising from 4.7 to 8.5% in adults [2]. About three million diabetic patients have been estimated, especially by 2025 [3]. In Europe, about 60 million people are affected by diabetes, with 90% estimated to have type 2 diabetes [4]. In Italy, diabetes prevalence has dramatically increased from 2.9% in 1980 to 5.3% in 2016 [5]. This increasing prevalence is strictly associated with an unhealthy diet, physical inactivity, and obesity, which are possible risk factors for increases in glucose serum levels. Glucose excesses can lead to extended cellular damage, with the consequence of several infectious and non-infectious skin disorders [1]. Among complications of diabetes, nail and skin infections represent a common condition. Peripheral neuropathy, compromised peripheral circulation and consequent decrease of foot sensation, place diabetics’ feet at high risk of trauma. This fragility condition predisposes the nail and its nail matrix to the entry of microorganisms. Literary and clinical data highlight an increasing rate of fungal infections, with a documented possible role in health complications in critically ill patients, such as diabetic ones. In those patients, onychomycoses represent almost 50% of nail disorders and damages, contributing to secondary bacterial infections, necrosis and risk of toe or lower leg amputation [6,7,8]. According to prevalence studies, approximately one third of diabetic patients suffer from onychomycosis [9,10,11]. Diabetes is considered one of the most important predisposing factors for onychomycosis, which reaches a percentage equal to 31.5% in this group, despite low rates shown by healthy patients for the same clinical condition. Diabetic patients present several clinical conditions favouring the development of onychomycoses. High levels of glycated haemoglobin (HbA1C) and prolonged diabetic status lead to nail thickness and acceleration of subungual keratinization. Such conditions increase the probability of onychomycoses [12,13]. According to the same literary data, being male and older is also a condition favouring onychomycoses. The nails of elderly subjects are frequently affected because of their slow development compared to young subjects’ ones. This slowness contributes to a delay in the replacement of infected nail portions. Structural changes or altered immunological responses can contribute to cutaneous diseases in elderly patients compared with the general population [13,14].

Depending on nail localization, different onychomycoses clinical conditions exist. Distal and lateral subungual onychomycoses, superficial onychomycoses, proximal subungual onychomycoses, endonyx onychomycoses and total dystrophic onychomycoses are major clinical forms [15]. Each onychomycosis condition accounts for typical clinical features (Table 1). 

Onychomycoses aetiological agents are mainly anthropophilic dermatophytes, especially *Trichophyton rubrum* and *Trichophyton mentagrophytes* var. *interdigitale*. Non-dermatophytic-moulds (NDMs) such as *Scopulariopsis brevicaulis* or *Aspergillus* species can also be involved in onychomycoses aetiology as primary or secondary pathogens. NDMs occasionally isolated from affected nails include *Fusarium* spp., *Acremonium* spp., *Alternaria* spp. and *Neoscytalidium* species. NDMs worldwide prevalence has a rate of 10–15% in onychomycosis aetiology. Yeast isolates, such as *Candida albicans* and *Candida parapsilosis* complex, can be considered the third cause of nail fungal infection. Their presence is mainly related to predisposing factors such as immunosuppression and diabetes [13]. It is essential to clinical recognize potential onychomycosis. Differential diagnoses include traumatic onycholysis and nail psoriasis, which can share some clinical features with onychomycosis. Numerous abnormalities along toenails are not mycotic, so microbiological tests are required to clarify an infectious source [15].

Laboratory-based diagnosis provides early confirmation of the onychomycoses clinical suspicion, accurately identifying the etiologic agents. The onychomycoses diagnosis can be conventionally made by direct microscopy, adding potassium hydroxide to microscopic slides, and fungal culture. However, conventional methods have a low to moderate sensitivity [15]. Furthermore, culture-based detection methods are often associated with a long turnaround time (TAT) and a negativity rate of 20–30% in the case of positive microscopy. Different molecular methods can report the presence of dermatophytes in case of nail infection. Their introduction allowed the identification of dermatophytes in nail plate fragments, confirming the diagnosis of onychomycoses, especially in those patients who underwent previous antifungal treatments without any clinical improvement [16]. 

A resolutive antifungal treatment for onychomycoses includes either pharmacological aspects or correct foot management. Pharmacological agents can be local or systemic: the choice strictly depends on several factors. Topical treatments are generally suitable in case of less than two nail plate folds involved in the infection development. Otherwise, the infection of more than two nail plate folds requires systemic antifungal treatment. Moreover, other clinical aspects of the infection are also considered: the involvement of less than 50% of the nail plate structure leads to topical treatment, whereas the affection of more than 50% of the nail plate structure leads to different therapeutical choices. Systemic treatments are often limited due to drug interactions and toxicity, while topical treatment can present low efficacy due to inefficient nail debridement. Combinations of treatments (topical plus systemic treatment) and nail thinning strategies are required to obtain better results in a short time and avoid relapses. Onychomycoses relapses are common when negative prognostic factors such as extended nail plate involvement, hyperkeratosis, white-yellowish stripes and dermatophytoma are present [15]. Timely treatment of onychomycosis is an additional aspect of diabetic foot care, potentially decreasing the risk of amputation. It seems relevant to investigate the frequency and the distribution of onychomycosis in diabetic patients, highlighting the diffusion of these infections and their potential role in a general worsening of a critical patient. 

The present study aimed to evaluate the toenail onychomycosis incidence in diabetic patients and healthy ones. A target was also to specify which species were more involved in the aetiology of onychomycosis in our geographical area. 

## 2. Materials and Methods

### 2.1. Patients and Methods

A non-interventional, retrospective study was performed at the mycology laboratory of the University hospital Policlinico-San Marco in Catania, Italy, for over one year (from January 2016 to December 2016) in the patient setting with suspected feet onychomycosis. The suspicion of onychomycosis is derived from the observations of one or more of the following signs: nail thinness, lifting of the nail plate, nail distortion, nail opacification and surrounding tissue inflammation. Information about age, sex, presence of diabetes and preceding antifungal treatment were recorded. Any type of antifungal treatment was suspended three months before nail sample collection. According to the aim of the study, two groups of patients with suspected onychomycosis were considered. 

Suspicion-infected nails were firstly hygienized with 75% alcohol. Then, a nail clipping was collected using a single-use sterile surgical scalpel. For microscopy examination, the nail clippings were settled on slides. Finally, a drop of 10–15% KOH was added, and a coverslip was used to firmly fix the slide. Slides were observed after an incubation of 45 min–1 h at the temperature of 25 °C to ensure a sample clarification. Cultures were performed using Sabouraud dextrose agar (SDA, Biolife, Milan, Italy) + chloramphenicol and +/− cycloheximide. Agar plates were incubated at 32 °C for up to 2 weeks and daily monitored for the possible growth of fungal colonies.

All mould isolates were firstly identified by standard phenotypic methods based on the macroscopic study of SDA colonies and Czapek agar medium (Difco, Becton Dickinson, Buccinasco, Italy) subcultures. Further characteristics were investigated through slide microcultures on Potato dextrose agar, which allows conidiogenesis. A small piece of this culture medium was cut and placed into a sterile Petri plate. Then, spots of colonies were located at the four angles of the medium fragment, using a sterile needle. Either aerial or vegetative mycelium was involved in this collection. A sterile coverslip was placed on the inoculated medium, which was incubated for 7–10 days at 25 °C. Finally, the coverslip was detached and placed on a new slide with a drop of blue-lactophenol. Microscopic identification was performed using conventional dichotomous keys. Yeasts were identified by cornmeal agar medium with the addition of 0.5% of Tween-80, according to the Dalmau plate technique. In addition, a germ tube test was performed to help identify fungal isolates. A germ tube test can be easily made through an inoculum of one suspected yeast colony in 0.5 mL of bovine fetal serum. The inoculated suspension can be incubated at 37 °C for up to 2 h and 30 min. A small amount of the suspension is placed into a slide and microscopically evaluated. This observation allows a presumptive yeast identification. Definitive identifications were obtained through analysis of biochemical patterns by ID32C kit (bioMérieux, Marcy étoile, France), which includes 32 biochemical assimilation tests.

Onychomycosis cases were defined as the combination of clinical evidence of nail disorder and positive microscopic and/or culture results, according to gold standards for diagnostic confirmation of nail fungal infection.

### 2.2. Statistical Analysis

Data were analyzed using the MedCalc Statistical Software version 17.9.2 (MedCalc Software bvba, Ostend, Belgium; http://www.medcalc.org, accessed on 18 July 2022). Medians with ranges were used to describe non-normally distributed continuous variables and compared using the Mann–Whitney U-test. Categorical variables are reported as percentages and compared using the two-tailed χ^2^ test or Fisher’s exact test, as appropriate.

## 3. Results

A total of 715 patients (47 diabetic and 668 non-diabetic patients) were enrolled. About 44.7% of patients underwent a previous empirical antifungal treatment. Among diabetic patients, nine underwent systemic antifungal treatment, while two used combined treatment and three used topical treatment. Among non-diabetic patients, 50 underwent systemic treatment, whereas 15 used combined therapy and 241 used topical treatment.

Systemic antifungal drugs frequently prescribed were fluconazole (49.9%), itraconazole (25.2%) and terbinafine (24.9%), while topical antifungals used were ciclopiroxolamine (40.2%), amorolfine (34.3%) and tioconazole (25.5%). A total of 493 patients were women (68.9%), while 222 were men (31.1%), with a median age of 56 years (95% CI: 54–58).

Patients’ demographic characteristics and onychomycosis prevalence in the two groups are summarized in Table 2. Specifically, age (*p* < 0.0001), as well as the onychomycosis prevalence (*p* < 0.0001), were significantly higher in the group of diabetic patients.

Regarding the fungal species involved in onychomycosis, a significant difference emerged between the two groups of patients (*p* = 0.0001). Specifically, in diabetic patients, dermatophytes were the most common cultural isolates (50%), followed by yeasts and moulds in 30.8% and 19.2%, respectively. In non-diabetic patients, the distribution of dermatophytes, yeasts and non-dermatophytic moulds was 67.4%, 5.3% and 27.3%, respectively (Table 3).

In both groups, dermatophytes were the most common fungal pathogens isolated from positive cultures of toenails samples. The spectrum of fungal pathogens isolated from diabetic patients was as follows: *Trichophyton mentagrophytes* was the most frequently isolated dermatophyte mould (38.5%), followed by *Trichophyton rubrum* (11.5%); *Penicillium decumbens* was the prevalent non-dermatophyte mould isolated (7.7%), followed by *Aspergillus nidulans* (3.8%), *Fusarium oxysporum* (3.8%) and *Scopulariopsis brevicaulis* (3.8%); the most frequent yeast species isolated were: *Candida albicans* (11.5%), followed by *Can-dida parapsilosis* complex (7.7%), *Candida guilliermondii* (7.7%), and by *Trichosporon cutaneum* (3.8%). Otherwise, in non-diabetic patients without the spectrum of fungal pathogens isolated was as follows: *Trichophyton rubrum* was the most frequently isolated dermato-phyte mould (38.5%), followed by *Trichophyton mentagrophytes* (27.2%) and *Microsporum canis* (1.8%); *Aspergillus* sp. was the prevalent non-dermatophyte mould isolated (13.6%), followed by *Scopulariopsis* sp. (9.4%), *Fusarium* sp. (1.8%), *Penicillium decumbens* (1.2%) and *Paecilomyces* sp. (1.2%); the most frequent yeast species isolated was *Candida parapsilosis* complex (2.9%), followed by *Candida albicans* (1.2%), *Candida guilliermondii* (0.6%), and by *Trichosporon mucoides* (0.6%). All the different isolated species are summarized in Table 4. 

## 4. Discussion

Undoubtedly diabetes is a significant risk factor for skin and nail disorders [8]. Onychopathies such as onychomycoses are common in critically ill patients. Consequently, fungal pathogens must be monitored for possible related health complications. For non-diabetic patients, nail infection problems are only correlatable to aesthetic issues. Otherwise, diabetic patients could undergo severe complications due to a nail infection, especially when misdiagnosed or recognized late. These consequences are possible because of a non-prompt therapeutic intervention. Prevalence data about superficial fungal infection in diabetic patients illustrate conflicting details. Some studies do not highlight any difference between diabetic and non-diabetic patients, while other articles estimate a higher onychomycoses incidence in diabetic patients [17,18,19]. Our data clarify that onychomycoses prevalence is highly significant in diabetic patients (*p* < 0.0001) compared with healthy ones.

Onychomycoses are generally considered diffused nail plate infections caused by dermatophytes, yeasts and non-dermatophytic moulds. They often infect fingers and mostly toenails. The most common etiological agents of toenail onychomycosis are dermatophytes species accounting for the highest percentage of onychomycoses cases. *Trichophyton rubrum* and *Epidermophyton floccosum* are mainly isolated in the case of onychomycoses. However, other possible causative agents are *Trichophyton interdigitale* and *Trichophyton tonsurans*. *Microsporum* spp. is rarely related to onychomycoses episodes. They often affect interdigital foot areas and extend their invasion to nails.

Dermatophytic ungual infections begin on the leading free edge of the nail plate or the lateral nail plate. They continue until the entire nail plate and bed are affected because adhesivity is promoted by fibrils which anchor fungal cells to host keratinocyte membranes.

Dermatophytes invade the overlying nail plate, detaching and distorting it after the adhesion phenomenon. They form hyphae which grow in several directions, entering the deeper stratum of the nail plate and contributing to the possible destruction of subungual areas. Invasion phenomena are facilitated by proteases, which are produced by fungal cells to destroy extracellular matrix proteins such as keratin and collagen. Their breakdown provides nutrients for invading fungi. According to literary data, susceptibility to dermatophyte nail infections could depend on transmission in households where affected members live. Frequent actions such as sharing slippers or walking on carpets or floors increase nail dermatophytoses transmission risk up to 44–47%. Furthermore, studies have suggested that dermatophytes are able to colonize textiles and containers: transmission risk seems to be high also due to environmental factors [20,21].

Possible aetiological agents of onychomycoses are non-dermatophyte moulds such as *Scopulariopsis brevicaulis, Fusarium* and *Aspergillus* species [21]. Onychomycoses due to Fusarium species come up with one single nail usually marked by a small white stain on the free edge of the nail plate or in the proximal region of the nail. This stain tends to extend to all the nail plates, which become opaque and whitish [22]. Onychomycosis due to *Aspergillus* spp. is usually a distal–lateral subungual onychomycosis and can lead to thick, brittle or discoloured nails [23]. *Scopulariopsis* species are the leading non-dermatophytic moulds causing onychomycosis.

*Scopulariopsis brevicaulis* causes brownish-yellow discolouration and hyperkeratosis. This species represents 1–10% of non-dermatophytic isolates, with variable incidence rates depending on geographical distribution, population features and diagnostic methods [24]. *Penicillium* and *Paecilomyces* species are rarely involved in human onychomycosis. They need a previous keratin alteration on the nail plate, so they are occasionally isolated from patients with preceding dermatophytic infection. The dermatophyte mould, in fact, weakens the nail keratin making it more prone to environmental mould invasion [25]. Yeasts are occasionally involved in the aetiology of onychomycosis. *Candida* species are the leading yeast cause of onychomycosis, which stands for greenish-black discolouration of the nail and possible onycholysis. The toenail onychomycosis aetiology in diabetic patients appears similar to that of the general population, but a recent study found an increased prevalence of *Candida albicans* in these patients’ onychomycoses [26]. According to current literary data, the pathogenic role of the *Trichosporon* genus in onychomycosis is not definitely confirmed. However, there are few reports about its single presence in patients with nail lesions. Some studies propose its possible role in human nail infections [27].

Our study highlights a statistically significant difference (*p* = 0.0001) considering fungal species involved in onychomycoses aetiology. Specifically, healthy subjects hold high percentages of dermatophytic isolates (67.4%), followed by NDM (27.3%) in onychomycoses aetiology. In diabetic patients, dermatophytic moulds reached a rate of 50%, followed by a consistent percentage of yeast isolates (30.8%) in onychomycoses aetiology. According to literary data, yeasts can be considered a common isolate in diabetic nail infection cases. *Candida* species have been identified as the second most common isolate after dermatophytic moulds [13].

The high yeast rate was explained considering that *Candida* species have a place in the ulcerated anatomical regions and interdigital feet zones. A yeast infection often spreads from these areas, extending to the perionychium and affecting the nail plate. 

Furthermore, literary data support an increase of yeast isolates in diabetic patients with onychomycosis [28,29]. Many authors evaluated that the growth rate of *Candida* species can be related to the hyperglycemic condition of a diabetic patient. Specifically, the glucose blood level is directly proportional to *C. albicans* growth, explaining how yeast infections are more common in uncontrolled diabetic patients than in healthy ones [13]. Moreover, *Candida* spp. has virulence mechanisms helping the pathogenesis of onychomycoses. For instance, this genus usually produces extracellular hydrolases such as proteinases, phospholipases and lipases. All of these hydrolases help tissue invasion.

*Candida* extracellular proteinases are produced to a greater degree when the substrate is enriched with keratin, such as human epithelium. These considerations show that keratin acts as an ideal growth environment for *Candida* strains, which progressively alter the expression of human defensin, leading to further invasion processes. It is also important to clarify that *C. albicans* is able to produce melanin particles during tissue infections: melanin probably protects fungal cells from antifungals and temperature. Several *Candida* species show a significant biofilm-forming ability, which contributes to development of ulcers, skin, and nail infections. In conclusion, multiple virulence mechanisms seem to be involved in *Candida* nail infections [13,26].

Non-diabetic patients present a low rate of nail fungal infections, among which dermatophytes onychomycoses caused by *Trichophyton* species hold the highest rate. For non-diabetic patients, non-dermatophytic moulds carry a medium-low incidence rate; however, it is essential to highlight their more varied species distribution compared to the diabetics’ group. In this group of patients, yeast isolates show the lowest percentage in onychomycoses aetiology. It was also essential to report geographical distribution mentions of aetiological agents.

According to literary data, dermatophytes are not always cosmopolitan in their world distribution. Some species have been detected from all the continents, while others are typically present in limited areas of the world.

In our geographical zone, onychomycosis is mainly caused by dermatophytic moulds, regardless of the type of selected patient. Other aetiological agents, such as non-dermatophytic-moulds and yeasts, are distributed differently depending on the type of patient.

Finally, significant differences emerged regarding a previous antifungal treatment which always has a negative impact on laboratory diagnostic processes. Specifically, healthy patients showed a high incidence of antifungal molecule use without a correct microbiological diagnosis or a clinical evaluation. Otherwise, diabetic patients tended to use less empirical antifungal treatment and often explain their symptoms to clinicians.

Finally, according to our data, diabetic patients are more predisposed to nail fungal infection due to several risk factors such as high glucose serum levels, vascular diseases, and a decline in foot sensation. It has been established that a diabetic patient has a highly fragile condition predisposing them to nail or skin infections. Consequently, breaking the household’s transmission cycle of assured pathogens such as dermatophytes should be a possible solution to preserve critically ill patients from infectious complications. Recommendations such as floor disinfection, minimizing shared personal tools, washing of textiles at high temperatures (60 °C) and maintaining pets’ hygiene should be applied.

Moreover, our data suggest that dermatological follow-ups should always be performed for diabetic patients. This process could allow a correct prediction and monitoring of plausible risk factors or infection sources. Skin and nail disorders should be carefully monitored to provide diagnostic confirmation and adequate management of diabetic patients. All these precautions could limit onychomycoses spread and transmission but also avoid re-infection processes in critical patients.

## Figures and Tables

**Table 1:** Clinical features of onychomycoses different types.

Onychomycoses Clinical Type	Clinical Features
Distal and lateral subungual onychomycoses	Changes to white-yellow in nail plate colour; predisposition to onycholysis and hyperkeratosis; high prevalence in toenails.
White superficial onychomycoses	Presence of small, white and dull areas which involve only the superficial fragment of the nail plate. Strong correlation between this condition and feet athlete’s syndrome or alterations in the posture of the toes.
Proximal subungual onychomycoses	Initial involvement of the nail matrix and subsequent invasion of the nail plate. Presence of a whitish area on the lunula, especially on the hand’s nails.
Endonyx onychomycoses	Extended nail plate invasion, without any involvement of the nail bed. Discolouration and lamellar splitting on affected nails.
Total dystrophic onychomycoses	The final stage of all onychomycoses clinical types. Presence of nail thickening, opacification and fissuring. The nail plate appears to be fragile and potentially destroyed.

**Table 2:** General characteristics of patients enrolled in the study.

Patients Characteristics	Total (*n* = 715)	Non Diabetics (*n* = 668)	Diabetics (*n* = 47)	*p*
Women (%)	493 (68.9)	467 (69.9)	26 (55.3)	0.036
Age, median years (95% CI)	56 (54–58)	54 (53–56)	70 (66–75)	<0.0001
Antifungal treatment (no., %)	320 (44.7)	306 (45.8)	14 (29.8)	0.032
Onychomycosis (no., %)	195 (27.3)	169 (25.2)	26 (55.3)	<0.0001

**Table 3:** Mycological findings in diabetic and non-diabetic patients.

Patients	Dermatophytes (*n* = 127)	NDM * (*n* = 51)	Yeast (*n* = 17)	p **
Diabetics (no., %)	13 (50)	5 (19.2)	8 (30.8)	0.0001
Non-diabetics (no., %)	114 (67.4)	46 (27.3)	9 (5.3)	
Total (no., %)	127 (65.1)	51 (26.2)	17 (8.7)	

* Non-dermatophytic molds. ** *p* values were calculated by summing the fungal isolates in diabetic and non-diabetic patients.

**Table 4:** Different isolated species in diabetic and non-diabetic patients.

Fungi	Patients	
	Diabetics	Non-Diabetics
**Dermatophytes (no., %)**		
*Trichophyton rubrum*	3 (11.5)	65 (38.5)
*Trichophyton mentagrophytes*	10 (38.5)	46 (27.2)
*Microsporum canis*	-	3 (1.8)
Subtotal	13 (50)	114 (67.4)
**Non-dermatophytic molds (no., %)**		
*Aspergillus nidulans*	1 (3.8)	-
*Aspergillus sydowii*	-	7 (4.1)
*Aspergillus flavus*	-	6 (5.5)
*Aspergillus niger*	-	4 (2.4)
*Aspergillus candidum*	-	3 (1.8)
*Aspergillus terreus*	-	3 (1.8)
*Fusarium oxysporum*	1 (3.8)	1 (0.6)
*Fusarium solanine*	-	2 (1.2)
*Penicillium decumbens*	2 (7.7)	2 (1.2)
*Paecilomyces variotii*	-	1 (0.6)
*Paecilomyces lilacinus*	-	1 (0.6)
*Scopulariopsis brevicaulis*	1 (3.8)	9 (5.3)
*Scopulariopsis fusca*	-	7 (4.1)
Subtotal	5 (19.2)	46 (27.3)
**Yeasts (no., %)**		
*Candida albicans*	3 (11.5)	2 (1.2)
*Candida parapsilosis*	2 (7.7)	5 (2.9)
*Candida guilliermondii*	2 (7.7)	1 (0.6)
*Trichosporon cutaneum*	1 (3.8)	-
*Trichosporon mucoides*	-	1 (0.6)
Subtotal	8 (30.8)	9 (5.3)

## Data Availability

All data generated or analyzed during this study are included in this published article.
